# A Novel SOD1 Intermediate Oligomer, Role of Free Thiols and Disulfide Exchange

**DOI:** 10.3389/fnins.2020.619279

**Published:** 2021-02-18

**Authors:** Bon-Kyung Koo, William Munroe, Edith B. Gralla, Joan Selverstone Valentine, Julian P. Whitelegge

**Affiliations:** ^1^Department of Chemistry and Biochemistry, University of California, Los Angeles, Los Angeles, CA, United States; ^2^The Pasarow Mass Spectrometry Laboratory, David Geffen School of Medicine, NPI-Semel Institute for Neuroscience and Human Behavior, University of California, Los Angeles, Los Angeles, CA, United States

**Keywords:** fibrillation, disulfide reduction, fibril seeding, Cu/Zn superoxide dismutase 1, amyotrophic lateral sclerosis, oligomerization, analytical ultracentrifugation

## Abstract

Wild-type human SOD1 forms a highly conserved intra-molecular disulfide bond between C57-C146, and in its native state is greatly stabilized by binding one copper and one zinc atom per monomer rendering the protein dimeric. Loss of copper extinguishes dismutase activity and destabilizes the protein, increasing accessibility of the disulfide with monomerization accompanying disulfide reduction. A further pair of free thiols exist at C6 and C111 distant from metal binding sites, raising the question of their function. Here we investigate their role in misfolding of SOD1 along a pathway that leads to formation of amyloid fibrils. We present the seeding reaction of a mutant SOD1 lacking free sulfhydryl groups (AS-SOD1) to exclude variables caused by these free cysteines. Completely reduced fibril seeds decreasing the kinetic barrier to cleave the highly conserved intramolecular disulfide bond, and accelerating SOD1 reduction and initiation of fibrillation. Presence or absence of the pair of free thiols affects kinetics of fibrillation. Previously, we showed full maturation with both Cu and Zn prevents this behavior while lack of Cu renders sensitivity to fibrillation, with presence of the native disulfide bond modulating this propensity much more strongly than presence of Zn or dimerization. Here we further investigate the role of reduction of the native C57-C146 disulfide bond in fibrillation of wild-type hSOD1, firstly through removal of free thiols by paired mutations C6A, C111S (AS-SOD1), and secondly in seeded fibrillation reactions modulated by reductant tris (2-carboxyethyl) phosphine (TCEP). Fibrillation of AS-SOD1 was dependent upon disulfide reduction and showed classic lag and exponential growth phases compared with wild-type hSOD1 whose fibrillation trajectories were typically somewhat perturbed. Electron microscopy showed that AS-SOD1 formed classic fibrils while wild-type fibrillation reactions showed the presence of smaller “sausage-like” oligomers in addition to fibrils, highlighting the potential for mixed disulfides involving C6/C111 to disrupt efficient fibrillation. Seeding by addition of sonicated fibrils lowered the TCEP concentration needed for fibrillation in both wild-type and AS-SOD1 providing evidence for template-driven structural disturbance that elevated susceptibility to reduction and thus propensity to fibrillate.

## Introduction

Over 100 different mutations in SOD1 (an abundant copper- and zinc-containing superoxide dismutase 1), the antioxidant enzyme, have been identified to account for fALS (familial amyotrophic lateral sclerosis), a fatal neurodegenerative disorder caused by formation of abnormal protein aggregates in neuronal cells. Detergent-resistant aggregates isolated from the spinal cords of ALS transgenic mice contain full-length apo hSOD1 proteins that acquire toxic properties in the disease mechanism (Shaw et al., 2008), and SOD1 fibrils have been shown to induce cytokine expression in mononuclear cells, thus causing inflammation (Fiala et al., 2010), and to activate microglial cells (Roberts et al., 2013), suggesting that fibrils have toxic properties that may be related to ALS. On the other hand, the Dokholyan group recently found evidence that SOD1 trimer is toxic to the type of neuron affected in ALS (Fay et al., 2016) and that large SOD1 fibrils protect rather than harm neurons (Zhu et al., 2018). However, still the fibrillation mechanism of SOD1 has remained elusive unlike that of other well-studied amyloids, and it is very important to understand the morphological differences and the reducing level of amyloid forming proteins in the cell of protein-misfolding diseases for comprehending of SOD1-fALS etiology.

SOD1 is a 32 kDa homodimer which forms a β-barrel and contains an intramolecular disulfide bond formed by C57 and C146 in addition to 2 reduced C6 and C111 in each 153-residue subunit (Valentine and Hart, 2003; Valentine et al., 2005). Over-expression of mutant SOD1 in neural tissues of fALS transgenic mice and in transfected cultured cells results in aggregation and neurotoxicity linked to inter-molecular disulfide cross-linking of the free cysteines C6 and C111 (Niwa et al., 2007; Cozzolino et al., 2008; Leinartaite and Johansson, 2013; Toichi et al., 2013). Protein fibrillary aggregation *in vitro* requires a significant conformational conversion of proteins thought to be first to the monomer (also known as “nucleus”), to subsequently form oligomers which is a rate-limiting step of the overall aggregation reaction (LeVine, 1999; Banci et al., 2006). Once the nucleus forms, it functions as “seed” (a structural template) to convert native dimeric proteins into β-sheet-rich monomeric structures and subsequently elongate the protein fibril. This mechanism, which accelerates and even triggers protein aggregation, is called the seeding reaction (Szedberg and Pedersen, 1940; LeVine, 1999; Bouldin et al., 2012). Protein conformational changes, aggregation and amyloid fibril formation can be monitored based on spectroscopic and biophysical methods such as fluorescence spectroscopy, analytical ultracentrifugation, and so on (Doucette et al., 2004; Cristovao et al., 2019).

The AS mutant, C6A/C111S, is a “pseudo-WT” SOD1 in which both free cysteines have been removed by mutation, with the buried Cys6 mutated to alanine and the surface Cys111 changed to serine. AS-SOD1 (mutant SOD1 lacking free sulfhydryl groups C6A/C111S) has a stability similar to that of WT-SOD1 but melts reversibly, while WT-SOD1 melts irreversibly, presumably because of disulfide-induced aggregation following thermal unfolding (Lepock et al., 1990; Hallewell et al., 1991; Parge et al., 1992; Oztug Durer et al., 2009). Here, we compared the seeding effect in the fibrillations of apo WT-SOD1 and apo AS-SOD1 to exclude variables caused by disulfide cross-linking of the free cysteines and to study the mechanism behind fibrillation lag time. During the lag time of SOD1 fibrillation, the protein undergoes conformational changes from native dimer to monomer which we further investigate by analytical ultracentrifugation. For this conformational conversion, the well conserved intramolecular disulfide bond of SOD1 requires reduction. When 10% of fully reduced fibril seed were added to the SOD1 fibrillation reaction, SOD1 reduction and fibrillation initiation was accelerated. We suggest some chaotropes such as low concentrations of GuHCl accelerated the fibrillation initiation possibly through a similar mechanism. Therefore, the mechanism behind decreasing the lag time is due to disulfide reduction and a conformational change that leads to accelerated initiation of fibrillation.

## Materials and Methods

### SOD1 Expression and Purification

Wild-type SOD1 was expressed in *S. cerevisiae* and purified following the procedures from the published procedures (Wiedau-Pazos et al., 1996; Doucette et al., 2004; Hough et al., 2004). C6A/C111S SOD1 (AS-SOD1) was expressed in *E. coli* and purified using a similar procedure. Purified SOD1 was demetallated by dialysis in a Slide-a-lyzer (Pierce, 10,000 Da. MWCO) against (1) 10 mM EDTA, 100 mM sodium acetate, pH3.8, (2) 100 mM NaCl, 100 mM sodium acetate, pH 3.8, and (3) 10 mM potassium phosphate, pH 7.0 as the dialysis buffer. Apo-SOD1 was flash-frozen in liquid nitrogen and stored at −20°C prior to use. Metal content of apo and metallated SOD1 was determined by inductive coupled plasma mass spectrometry (ICP-MS, Agilent 7,500 Series). All apo-proteins contained less than 0.1 equivalents of Cu and Zn per dimer.

### *In vitro* Fibril/Fibril Seed Formation

To make fibrils, 40 μM of apo protein was prepared in 10 mM potassium phosphate (KPi), pH 7.0 with 5 mM TCEP and 40 μM of thioflavin-T (TfT) as described (Chattopadhyay et al., 2008). TfT binds to beta sheet-rich structures, such as those in amyloid aggregates, and enhances fluorescence, so it is often used as a diagnostic of amyloid structure (LeVine, 1999). The protein samples were reduced with 5 mM TCEP during fibrillation at 37°C without shaking. To monitor fibril formation, 200 μM of each reaction was in separate wells of a 96 well plate to which a Teflon ball (1/8 in. diameter) had been added. The plate was agitated at 300 rpm (3 mm rotation diameter) in a Fluoroskan plate-reader (Thermo) at 37°C. Fluorescence measurements were recorded every 15 min using at λ_*ex*_ = 444 nm λ_*em*_ = 485 nm, with an integration time of 200 ms. All reactions were performed in replicates of 3 or more. Data was fitted to the following equation:

(1) F=F+0(A+ct)/(1+exp[k(t-m-t)],

where lag phase was calculated as t_*m*_ – t.

Fibril seeds were prepared from fibrils generated as described above. Fibrils extracted from a 96-well microplate were centrifuged in a microcentrifuge tube at 16,800 *x* g for 15 min. The supernatant was pipetted out, washed with 10 mM potassium phosphate buffer twice and the fibrils were resuspended in the same volume (200 μL) of 2 M guanidinium hydrochloride, 10 mM potassium phosphate, pH 7, by pipetting up and down and vortexing at medium speeds and incubated at 37°C for 90 min. They were then moved to a glass vial and sonicated for 30 min in a water bath sonicator (Branson Scientific, 100 watts) and centrifuged to remove supernatant (guanidinium hydrochloride) twice and then used within 30 min. Seeding reactions were assembled using 10–20% by volume of fibril seeds prepared by the above protocol, 40 μM apo SOD1 and 40 μM ThT in 10 mM potassium phosphate, pH 7.

### Iodoacetamide Labeling

Disulfide-intact or disulfide-reduced apo-SOD1 at 3–5 mg/ml was treated with 20 mM IAA at room temperature for 1 h in dark. Excess IAA and TCEP were removed by 3 rounds of centrifugation in 10 mM potassium phosphate, pH 7. The presence of IAA bound to cysteines was checked by ESI-MS.

### Proteolysis of Fibrillar and Soluble SOD1

Trypsin (porcine; Promega) proteolysis was in 50 mM NH_4_HCO_3_ at 37°C for 30 min at 1:30 (wt:wt) enzyme:substrate. SOD1 fibrils were separated from soluble contaminants by ultracentrifugation in an Airfuge (Beckman Coulter) at 125,000 × g for 30 min; then the pellet was washed with 50 mM NH_4_HCO_3_. Soluble apo SOD1 was diluted to the same concentration as the fibril with 50 mM NH_4_HCO_3_ and was digested similarly. After proteolysis, the reaction was quenched by 1 mM PMSF.

### SEC-HPLC and ICP-MS

Disulfide-oxidized and -reduced apo-SOD1, which are dimeric and monomeric, respectively, were prepared and performed for SEC-HPLC and high-resolution MS/MS as previously described (Chattopadhyay et al., 2008; Chan et al., 2013). For the ICP-MS, 20 μL sample was taken and added 50 μL of nitric acid (Optima grade). Then, it was heated for 2 h at 95°C with opened lid. It was then diluted with 2 mL of 2% nitric acid and added 20 μL internal standard for ICP-MS. The experiment was performed as previously described (Chattopadhyay et al., 2015).

### Analytical Ultracentrifugation

SOD1 samples were examined by sedimentation velicity using a Beckman XL-A analytical ultracentrifuge equipped with the AN-60 rotor (Beckman Instruments). SOD1 samples were loaded into 1.2 cm path length charcoal-filled Epon double sector cells. Measurements were recorded at 280 nm to monitor the behavior of the SOD1 sample at various rotor speeds. For all the measurements, the sedimentation coefficient values were corrected to S_20__,w_ (standard solvent conditions in water at 20°C).

Hypothetical sedimentation coefficients for monomeric and dimeric SOD1 were calculated from the molecular weight, partial specific volume, and approximate axial ratio using the following equation:

(2) $$s=\frac{M^{2/3}(1-\bar{v}\text{ρ})}{N6\text{πη}{\left(\frac{3}{4\text{π}N}\right)}^{1/3}{\left(1+\frac{\text{δ}H}{\bar{v\text{ρ}}}\right)}^{1/3}\left(f/f_{0}\right)}$$

Where the terms are as follows: s is the sedimentation coefficient in seconds (with S having units of 1 × 10^–13^ s), M is the macromolecule’s molecular weight,$\bar{v}$ is the partial specific volume of the macromolecule, *ρ* is the solvent density, N is Avogadro’s number, *n* is the solution viscosity in Poise, *δH* is hydration of the macromolecule in g water per g macromolecule, and *f*/*f*_0_ is the calculated Perrin factor for the asymmetry of the macromolecule (Szedberg and Pedersen, 1940; Schachman, 1959; Tanford, 1961; Van Holde et al., 1998).

### Electron Microscopy

Five microliters of the fibril suspension were deposited on a formavar-coated copper grid (Ted Pella, Inc.). The sample was allowed to adsorb for 5 min, blotted, washed 3 times with 10 mM HEPES, blotted and then stained with freshly filtered 2% uranyl acetate (100 μM) for 3 min, blotted again. The grids were air-dried for 30 min before insertion into a JEM12000 electron microscope operated at 80 keV. Fibrils were typically visualized at a magnification of 75,000.

## Results

### Fibrillation of Apo AS-SOD1 Is Dramatically Changed Depending Only on the Reduction of s-s Bond

In addition to Cys-57 and Cys-146, which form the native intramolecular disulfide bond, SOD1 contains 2 reduced cysteine residues, Cys-6 and Cys-111, and during the lag time, we could observe disulfide scrambling between (Cys-111 and Cys-146) or (Cys-6 and Cys-111) (Supplementary Figure S1). Consequently, we wanted to compare WT-SOD1 to AS-SOD1, which is mutated C6A and C111S to exclude the effect of disulfide scrambling. Apo AS-SOD1 formed amyloid-like aggregates at neutral pH under reducing conditions as WT. To compare the fibrillation, we used fibrils generated by incubating apo WT^*S*–*S*^ or apo AS^*S*–*S*^ in the presence of varying concentrations of TCEP (previous lab studies used dithiothreitol).

**FIGURE 1:**
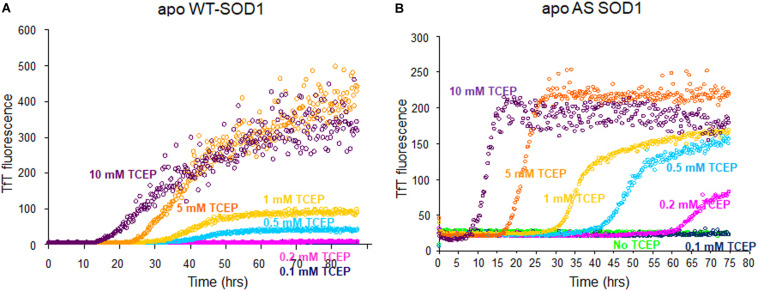
Fibrillation comparison of apo WT- (yeast) **(A)** and apo AS-SOD1 (*E. coli*) **(B)** in the various concentrations of TCEP. Apo WT SOD1 (40 μM) in 10 mM potassium phosphate buffer, pH 7.0 with additions as indicated was incubated with constant agitation at 37°C in 96-well plates. TfT fluorescence was monitored as an indicator of fibril formation. The monitored TfT fluorescence in the various concentrations of TCEP is shown in green (no TCEP), navy blue (0.1 mM), pink (0.2 mM), cyan (0.5 mM), gold (1 mM), orange (5 mM), and purple (10 mM), respectively.

The fibrillation of apo WT-SOD1 showed more complex behavior than apo AS-SOD1 (compare Figures 1A,B) because it has more variables such as disulfide scrambling. In fact, apo AS-SOD1 shows dramatically cleaner TfT fluorescence profiles. TfT fluorescence profiles of WT vs. AS-SOD1 depends on the TCEP concentrations (Figures 1A,B). Across the TfT fibrillation experiment, AS had shorter lag times than WT at comparable TCEP concentrations, and the slope of AS was consistently steeper during the growth phase compared with WT. The simplest interpretation is that reduction of the disulfide allows unhindered amyloid growth in AS whereas WT is delayed and slowed down by disulfide scrambling. The free C6 and C111 thiols are clearly un-necessary for fibrillation of AS-SOD1 in the presence of reductant and the process appears more efficient because disulfide scrambling is eliminated in the AS mutant.

### Reducing of Disulfide Bond During Lag Time

**FIGURE 2:**
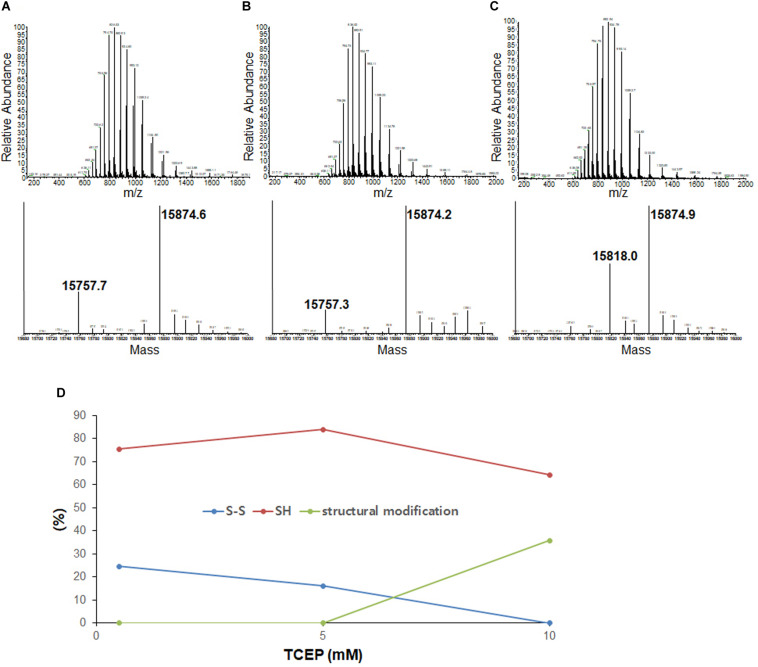
LC-MS of the lag phase of apo AS-SOD1 in 0.5 mM **(A)**, 5 mM **(B)**, and 10 mM **(C)** TCEP at 47 h **(A)**, 7.5 h **(B)**, and 5.5 h **(C)**, respectively. Molecular weights of AS S-S and AS SH treated with iodoacetamide are about 15756.43 Da and 15870.53 Da based on PeptideMass (http://web.expasy.org/cgi-bin/peptide_mass/peptide-mass.pl). **(D)** The percentages of the ratio of AS S-S and AS SH are shown in different TCEP concentrations.

To compare disulfide bond status around the initiation of fibrillation in different TCEP concentrations (before growth phase has started) we used mass spectrometry after iodoacetamide (IAA) treatment (if the disulfide is intact then the molecule is 112 Da lighter – 2 molecules IAA minus a disulfide). Samples were collected at 47 h in 0.5 mM TCEP, 7.5 h in 5 mM TCEP, and 5 h in 10 mM TCEP after shaking the 96-well plate right before the initiations. In the presence of 0.5 mM TCEP, separate peaks corresponding to apo AS^*SH*^ (Mw 15,870.53 Da) and apo AS^*S*–*S*^ (Mw 15,756.43 Da) were observed with a ratio of approximately 1.8–1. In the 5 mM TCEP, a single peak for the mixture of apo AS^*SH*^ and apo AS^*S*–*S*^ as the ratio of 7.5–1. However, in the 10 mM TCEP, it was a single peak of completely reduced apo AS-SOD1 (Figure 2). Fibrillation initiation is slowly started around 47 h if intact disulfide bonds are less than 25% (Figure 2D) in case of 0.5 mM TCEP (these ratios assume that disulfide reduction and modification of each thiol with IAA does not change the ionization efficiency).

Previously, we showed that NoCys mutant spontaneously fibrillates with a lag phase of about 10 h (Chattopadhyay et al., 2015), and apo NoCys-SOD1 has been shown to behave structurally and biophysically very close to WT^2S*H*^ (Furukawa and O’Halloran, 2005; Furukawa et al., 2008). This is comparable to a lag phase of about 15 h for apo WT^*S*–*S*^ or about 7 h for apo AS^*S*–*S*^ in 10 mM TCEP, the concentration high enough to completely reduce intact disulfide bond (Figure 2C). Although AS is completely reduced in 10 mM TCEP, we could see the mass of 15,818 Da, suggesting modification with one thiol-molecule IAA, possibly suggesting that some structural modification during the lag time renders one thiol somewhat in-accessible to the IAA (Figures 2C,D). All these experiments show that the substantial majority of all disulfide bonds is reduced before growth phase of fibrillation commences.

### Lowering the Kinetic Barrier by Amyloid Seeds

**FIGURE 3:**
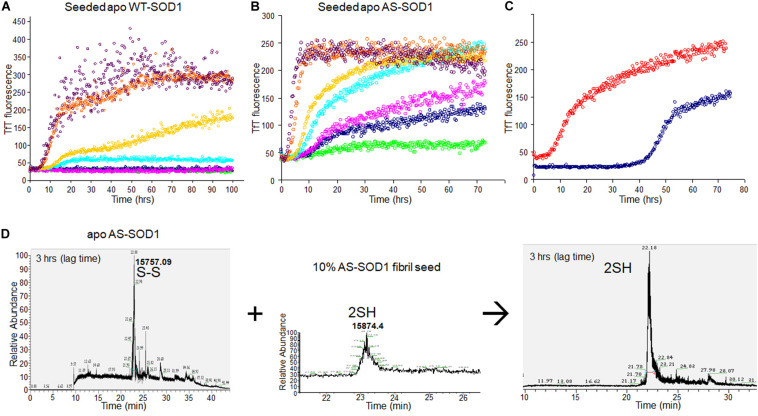
10% fibril seeds (AS-SOD1, see Figure 3) were added to the fibrillation of apo WT-SOD1 (40 μM) **(A)** and apo AS-SOD1 (40 μM) **(B)** in the various concentrations of TCEP, in 10 mM potassium phosphate buffer, pH 7.0. TfT fluorescence was monitored as an indicator of fibril formation. The monitored TfT fluorescence in the various concentrations of TCEP is shown in green (no TCEP), navy blue (0.1 mM), pink (0.2 mM), cyan (0.5 mM), gold (1 mM), orange (5 mM), and purple (10 mM), respectively. **(C)** Comparison of unseeded (navy blue) and 10% seeded (red) fibrillation of apo AS-SOD1 in 0.5 mM TCEP. **(D)** 10% fully reduced fibril seeds accelerated SOD1 reduction.

To examine the seeding effect, fibrils were generated by incubating apo WT^*S*–*S*^ or apo AS^*S*–*S*^ in 5 mM TCEP. The fibril from apo AS consists completely of AS^2S*H*^ in the presence of GdmCl, as shown in Supplementary Figure S2 by denaturation in the presence of IAA and subsequent HPLC-MS. It is mainly AS^2S*H*^ in the absence of GdmCl also (Supplementary Figure S2). When seeds prepared from these fibrils (AS^2S*H*^, absence of GdmHCl) were added at 10% to soluble apo WT SOD1^*S–S*^ and apo AS SOD1^*S–S*^ in the various concentrations of reducing agents, fibril growth for AS-SOD1 occurred dramatically with a very short lag phase compared to WT-SOD1 (Figures 3A,B). Strikingly, we found that addition of 10% seed decreased the necessary TCEP for the fibrillation initiation as well as it decreased the lag time (compare AS vs. WT; Figures 3A,B). Unseeded reactions did not fibrillate at 0.1 mM TCEP (Figure 1), but seeded reactions showed the fibrillation initiation at 0.1 mM (navy blue) or even in the absence of TCEP in case of apo AS-SOD1 (Figure 3B, green).

Comparing the TfT fluorescence at 0.5 mM TCEP of seeded vs. unseeded apo AS-SOD1, the lag time of unseeded fibrillation (compare with Figure 1) is around 40 h (navy blue), however, the lag time of 10% seeded fibrillation is shorter than 5 h (red) (Figure 3C). LC-MS was performed to examine the disulfide bond status in this seeding reaction. The fibrillated aggregation was initiated at 40 h without seed or 5 h with seed in 0.5 mM TCEP as shown in Figures 1, 3, therefore LC-MS was performed for the following reaction mixtures: (1) after 3 h shaking incubation, (2) fibril seed, and (3) 10% seed added reaction mixture after 3 h shaking incubation. We found apo AS-SOD1 in 0.5 mM TCEP is oxidized with intramolecular disulfide bond at the beginning of shaking incubation, but if 10% reduced fibril seed is added, it is also completely reduced rapidly (Figure 3D). Clearly, reduced fibril seed has a powerful ability to initiate fibrillation in the AS mutant.

### It Takes Time to Reduce Intact Disulfide Bond During Lag Time in Low TCEP

**FIGURE 4:**
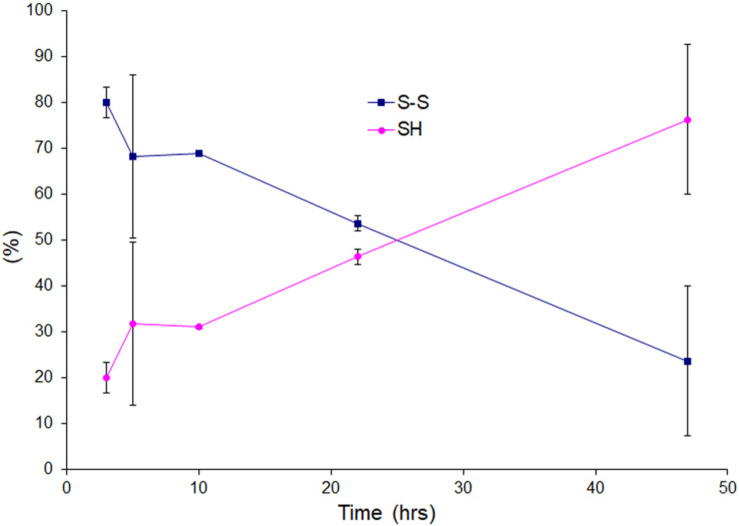
Oxidation and reduction of thiol group of the lag phase of apo AS-SOD1 in 0.5 mM TCEP at 3, 5, 10, 22, and 47 h.

We wanted to see the disulfide bond status during lag phase as time proceeds in presence of very little amount of reducing agent, so we performed LC-MS for reaction mixtures collected as time courses for apo AS-SOD1 thiol-alkylated by IAA in the low TCEP of 0.5 mM, and displayed the percentage of disulfide status as time passed (Figure 4). During the lag phase of apo AS^*S*–*S*^ fibrillation in 0.5 mM TCEP, it takes a long time of about 40 h to reduce the intact disulfide bond to initiate the fibrillation (Figure 1B, cyan), and the ratio of apo AS^*SH*^ to apo AS^*S*–*S*^ was approximately 1:10.3 at 3, 1:3.8 at 5, 1:4.8 at 10, 1:1.3 at 22, and 1:0.54 at 47 h (these ratios assume the ionization efficiency is unchanged by reduction). The initiation of apo AS-SOD1 is started when intact disulfide bond is reduced more than about 70% (Figures 2D, 4). However, when completely reduced 10% of apo AS-SOD1 fibril seeds were added to 5 mM TCEP fibrillation reaction, it could lower the lag time substantially suggesting lowering the kinetic barrier to onset of fibrillation (Figure 3D).

### Amyloid Fibril Images of Apo WT-SOD1 and Apo AS-SOD1

**FIGURE 5:**
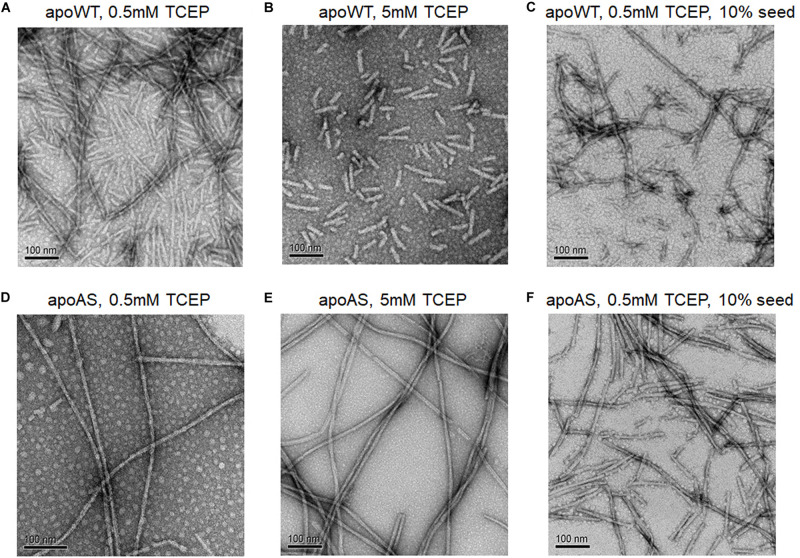
Electron micrographs of SOD1 fibrils. **(A)** apo WT-SOD1 fibrils in the presence of 0.5 mM TCEP **(B)** apo WT-SOD1 fibrils in the presence of 5 mM TCEP **(C)** 10% seeded apo WT-SOD1 fibrils in the presence of 0.5 mM TCEP **(D)** apo AS-SOD1 fibrils in the presence of 0.5 mM TCEP **(E)** apo AS-SOD1 fibrils in the presence of 5 mM TCEP **(F)** 10% seeded apo AS-SOD1 fibrils in the presence of 0.5 mM TCEP.

The electron microscopy (EM) confirmed the presence of amyloid fibrils and enabled comparisons of apo WT-SOD1 and apo AS-SOD1 fibrils (Figure 5). Apo AS-SOD1 fibrils (Figures 5D–F) look smoother like classical amyloid. Apo WT-SOD1 fibrils look sparse, and we can see the non-fibrillar intermediates of apo WT-SOD1 looking short with rounded ends (sausage-like) and lacking the dark characteristic of amyloid. Comparison of WT (5 mM TCEP; Figure 5B) vs. AS (5 mM TCEP; Figure 5E) shows the most dramatically contrasting comparison. Furthermore, WT (0.5 mM TCEP; Figure 5A) showing the sausage-like intermediates contrasting with mature amyloid fibrils in a single image is striking. It is also shown the globular soluble apo WT- or AS-SOD1 in the background as white spots. When 10% fibril seeds were added, it looks like soluble globular SOD1 proteins are budding on the various very short fibril fragments to longer fibril fragments (Figures 5C,F). We conclude the AS mutant fibrils move rapidly to form mature amyloid (classical amyloid appearance), whereas WT amyloid formation is disrupted allowing us to see what appear to be novel non-amyloid intermediates more easily.

### Conformational Change During Lag Time

**FIGURE 6:**
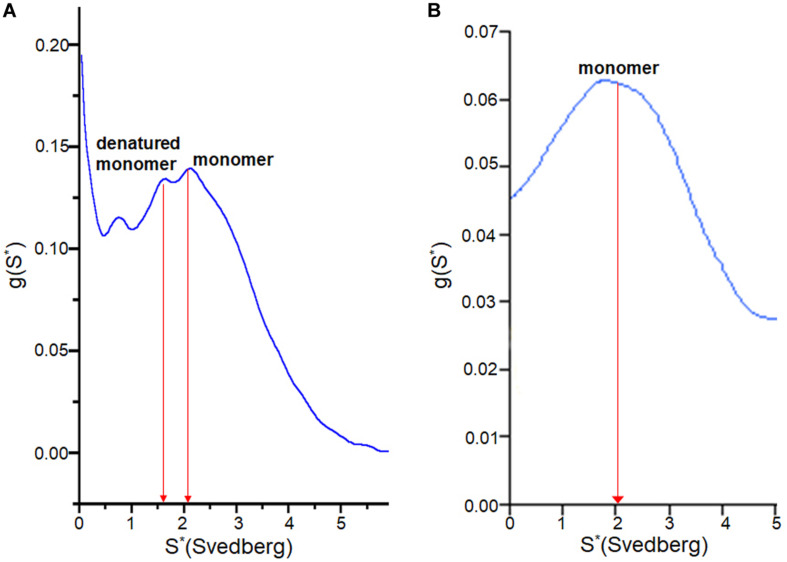
g(s) plot from sedimentation velocity of analytical ultracentrifugation on SOD1. It shows conformational change of apo WT-SOD1 for 10 h incubation **(A)** and apo AS-SOD1 for 12 h incubation **(B)** at 37°C in 5 mM TCEP during lag time of fibrillation. The hydrodynamic property (S_20__,w_) of SOD1 is about 2 for the monomer and about 3 for the dimer.

To examine how the TCEP treatments affected the SOD1 protein during fibrilization, sedimentation velocity experiments were performed using the soluble supernatant. The observed sedimentation coefficients of SOD1 measured during fibrillation lag time correspond to either monomer (apo AS-SOD1) or a partially denatured monomer (apo WT-SOD1). The reason the lag time of apo WT-SOD1 is longer than apo AS-SOD1 may be due to the fact it is the mixture of monomer and denatured monomer during the lag time of apo WT-SOD1, which requires more time to undergo more complex conformational conversions with four cysteines (Figure 6A), whereas apo AS-SOD1 is only monomer (Figure 6B).

### Guanidine Hydrochloride Behaves Similarly to Amyloidal Seeds

**FIGURE 7:**
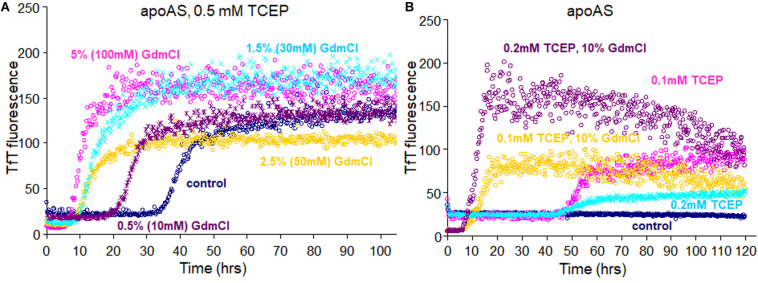
Fibrillation of apo AS-SOD1^*S–S*^ **(A)** in presence of 0.5 mM TCEP with addition of various concentrations of GuHCl and **(B)** with addition of 10% of GuHCl in 0.1 or 0.2 mM TCEP concentrations.

At the beginning SOD1 fibril was denatured in GuHCl for making amyloid seeds, but later we found that GuHCl can also affect SOD1 fibrillation at much lower concentrations. At 100 mM GuHCl the lag time decreased from about 40 h to about 5 h, like SOD1 fibril seeds in the presence of 0.5 mM TCEP (Figure 7A). Even 10 mM (0.5%) GuHCl reduced the lag time to almost half, about 18 h, presumably in a way that is quite distinct from the mechanism of protein denaturation at 6 M. As with 10% seeded apo AS-SOD1 fibrillation, 10% GuHCl also shortened the lag time even at 0.1 mM TCEP (Figure 7B). A reasonable comparison of seeding vs. low concentrations of chaotropes such as GuHCl in accelerating the reduction kinetics of SOD1 and promoting protein aggregation and fibrillation *in vivo* will bring us closer to understanding the precise molecular mechanism of fibrillation of SOD1.

## Discussion

Previously, our laboratory showed that monomeric apo, disulfide-reduced WT-SOD1, represented as apo WT^2S*H*^ (the unmodified polypeptide released from the ribosome before it acquires the earliest posttranslational modification), could initiate fibrillation of disulfide-intact forms of the protein (WT^*S*–*S*^), either apo or zinc-bound, at low, sub-stoichiometric amounts. This activity required the presence of thiols on cysteine residues at both positions 57 and 146, as single mutants apo-C57S or -C146S were unable to initiate fibrillation of apo WT-SOD1, nor were the S-alkylated form of apo, disulfide-reduced WT (Chattopadhyay et al., 2008). In addition to Cys-57 and Cys-146, which form the intramolecular disulfide bond, SOD1 contains 2 reduced cysteine residues, Cys-6 and Cys-111, and it is known that SOD1 undergoes disulfide scrambling with these reduced cysteines (Supplementary Figure S1; Leinartaite and Johansson, 2013; Toichi et al., 2013). We chose 0.5 mM TCEP concentration which is low enough to initiate SOD1 fibrillation (Supplementary Figure S1A) for cysteine alkylation and tryptic digestion before SOD1 fibrillation, and it is well supported by the mass spectrometry data where alkylation and tryptic digestion of apo SOD1 revealed the presence of peptides representing SOD1 with non-native intramolecular disulfides, including C111-C146 or C6-C111 (Supplementary Figure S1B) as presented by Leinartaite (Leinartaite and Johansson, 2013). So, we wanted to compare WT-SOD1 to simpler AS-SOD1 which is mutated C6A and C111S to exclude the effect of disulfide scrambling. In humans the initiating Met residue is removed and the second amino-acid residue is N-acetylated. Since human SOD1 was first sequenced as the protein form it has been traditionally numbered from this Ala residue as A1. A1 has not been found among hSOD1 mutations and N-terminal acetylation aparently does not affect amyloid behavior. We compared the fibrillation of WT-SOD1 from *Saccharomyces cerevisiae* (with N-terminal acetylation) and from *E. coli* (without N-terminal acetylation), and they showed almost identical fibrillation kinetics (data not shown). Consequently, we have used expression in *Saccharomyces cerevisiae* for WT-SOD1 because it is faithful to the acetylated N-terminus of hSOD1 and in *E. coli* for AS-SOD1 because it is easy to get overexpressed and quickly purified.

We found that the free Cys thiols are un-necessary for fibrillation (in the presence of reductant) as shown in Figure 1. Fibrillation of AS-SOD1 shows the initiation point and stationary point clearly better than WT-SOD1. It is initiated faster than WT-SOD1 in mostly reduced condition at 5 or 10 mM TCEP and started to initiate even in very little reduced condition at 0.2 mM TCEP in contrast with WT-SOD1. This indicates that disulfide scrambling may slow down and interfere with fibrillation, in some ways additional two free thiols in human SOD1 may be one of a range of protective mechanisms to retard the aggregation of SOD1, such as correct metal binding which is critical to prevent misfolding or tryptophan residue 32 in hSOD1 which is investigated as having a modulating role in its ability to propagate and template aggregation (Leal et al., 2015; Crown et al., 2020). This is the notable difference of SOD1 compared to other proteins such as α-synuclein, an intrinsically disordered protein, which are capable of fibrilization (Sarafian et al., 2017).

We varied TCEP concentration to see the effect on lag time of apo AS-SOD1. It was confirmed that the thiol groups of apo AS-SOD1 are more reduced at higher TCEP concentrations (Figure 2). Recently, it is reported that the disulfide bond of apo WT-SOD1 was reduced by 30% in 10 mM TCEP at 37°C, pH 7.4 based on differential scanning calorimetry (DSC) and size-exclusion chromatography (SEC-HPLC) (Abdolvahabi et al., 2016). This was compared with apo AS-SOD1 which was around 70% reduced in 10 mM TCEP at 37°C, pH 7.0 (Figure 2D) probably due to a slightly more favorable configuration. Once formed, the disulfide bond in WT hSOD1 is partially buried near the dimer interface (Banci et al., 2006). In fact, the accessibility of the disulfide bond may be a significant factor controlling the differences in disulfide reactivity of apo vs. holo, WT vs. ALS (or other) mutant hSOD1. The thermodynamic stability of the disulfide in ALS mutants is not consistently lower than WT hSOD1. However, the reduction rate of the disulfide is consistently faster for ALS mutants (Bouldin et al., 2012). Removal of all cysteines in SOD1 prevents formation of any disulfide linked aggregates, and the thiol-disulfide status in SOD1 is important in determining the aggregation state of the protein (Furukawa et al., 2004). Previously we used 5 mM DTT to form SOD1 fibrils, but later we found that DTT caused more oxidation on SOD1 during fibrillation based on MS data (data not shown). TCEP is not a thiol-containing reagent, so it performs no direct disulfide exchange function. TCEP is more powerful an irreversible reducing agent, more hydrophilic, and more resistant to oxidation in air. Since TCEP concentrations affect the lag time, this suggests that reduction of the disulfide bonds must precede fibrillation and it is the rate determining step of the fibrillation initiation of SOD1. However, complete reduction of intact disulfide bond is not exactly essential for the fibrillation initiation. Fibrillation initiation is slowly started at around 40 h if intact disulfide bonds are less than ∼30% in case of 0.5 mM TCEP (Figure 4). A conformational change is observed at higher than 5 mM TCEP (Figures 2C,D).

Misfolding/aggregation of mutant SOD1 is transmissible through a seeding mechanism inside the cell and among cells. Seeded aggregations of SOD1 proteins are a key event to understand progression/propagation of pathological changes in SOD1-related fALS and even sALS cases without mutation in SOD1 (Ogawa and Furukawa, 2014). For other proteins, it is known that the addition of fibril seeds to a solution shortens the lag time of the forming fibrils (Lee et al., 2007). This effect has been termed “nucleation-dependent” by Wood et al. and they explained that the added seeds act as catalytic sites that induce conformational changes in the protein (alpha synuclein) and accelerate fibrillation reaction rates (Wood et al., 1999; Scheibel et al., 2004). We wanted to examine the seed effect on both wild type and the simpler AS mutant of SOD1 fibrillations during the lag phase in detail to exclude the effect of disulfide cross linkage as we described above. Seeds are commonly generated by sonication driven shearing of mature fibrils to generate active ends (Furukawa and O’Halloran, 2005). They are then incubated with soluble protein and TfT fluorescence is monitored as a measure of the rate of fibril growth (Chattopadhyay et al., 2015). The key findings of our study are: (1) mostly reduced amyloid seed lowered the kinetic barrier of SOD1 fibrillation and accelerated amyloid fibrillation. Somehow seeds interact with monomeric apo SOD1 and render the native disulfide bond more exposed raising susceptibility to reduction, via an induced conformational change that improves accessibility to reductant. (2) AS-SOD1 shows kinetics clearly. The lag time of fibrillation is very long in low TCEP concentration (Figure 2, **60** h in 0.2 mM, 40 h in 0.5 mM), which means it takes a long time to reduce intact disulfide bond, but under more strongly reducing conditions such as 5 or 10 mM TCEP, its lag time is comparable to 10–20 h lag time of α-synuclein which does not have a disulfide bond. In 5 mM TCEP, mostly reduced condition, apo AS-SOD1 is monomeric, however, apo WT-SOD1 is a mixture of native monomer and partially denatured monomer based on sedimentation velocity experiments performed in the analytical ultracentrifuge (Figure 6). This indicates that apo WT-SOD1 has larger conformational complexity than AS-SOD1 resulting from disulfide scrambling using the two free thiols in WT-SOD1 that is not possible in AS-SOD1.

**FIGURE 8:**
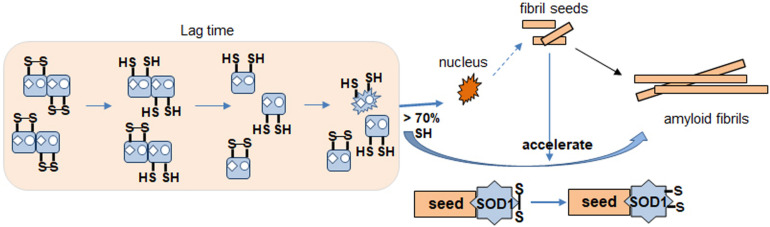
Proposed apo SOD1 fibrillation model. Formation of nucleus for fibrillation involves destabilization of dimer by metal loss. Disulfide reduction and monomerization allow conformational change to a species that is more likely to proceed toward fibrillation and amyloid formation (nucleus). Seed enhances kinetics of this reaction. Disulfide shuffling in WT but not mutant AS interferes with fibrillation causing a delay.

Based on our results identifying redox status of apo SOD1, we propose a model for the SOD1 fibrillation pathway (Figure 8). During lag time, SOD1 undergoes conformational changes from dimer to monomer, denatured monomer as its disulfide bond status changes. It is the rate determining step of the fibrillation initiation of SOD1, and human SOD1 has several controlling factors of this process like free thiols, metal binding or some specific residue like Trp32. The apo form of disulfide reduced SOD1 initiates to form the amyloid nucleus or seed if disulfide reduced forms are more than 70%. The monomeric nucleus subsequently becomes stabilized by recruitment of additional soluble WT or mutant SOD1 and elongates to form a mature fibril in multiple steps (Chattopadhyay et al., 2015). Seeded growth proceeds this way as well. The intact disulfide bond in SOD1 is buried and hard to break but somehow fibril seed is capable of increasing accessibility in order to break the dislfide faster, presumably by a conformational change.

Some chaotropes like GuHCl increase flexibility of SOD1, as do ALS mutations *in vivo*. It shows that GuHCl at low concentration can also affect the SOD1 fibrillation similar to amyloid seeds (Figure 7). Thus, mostly reduced SOD1 fibril seeds (Supplementary Figure S2) seem to associate with SOD1 molecules during lag time and act like a chaotropic denaturant. The EM showed immature intermediates of apo WT-SOD1 looking “sausage-like” quite distinguishable from mature fibrils of apo AS-SOD1 (Figure 5). It seems that alternative disulfide shuffling delays fibril maturation allowing us to view intermediates in the fibrillation process. Further study to investigate the toxicity of these intermediates compared to mature fibrils will be critical.

If amyloid formation is more efficient when the two free thiols are missing, then why has human SOD1 kept them given evolutionary pressure? One possibility lies in the intermembrane space (IMS) of the mitochondria as part of a quality control mechanism to identify faulty individual mitochondria. SOD1 has to lose its co-factors and structure for passage across the outer mitochondrial membrane and refolding in the IMS requires the chaperone Copper Chaperone for Superoxide dismutase (CCS). The correct disulfide has to be reformed and maybe the two free thiols provide complexity to the system such that elevated levels of activated oxygen (superoxide and hydrogen peroxide) damage the protein before it can fold correctly leaving misfolded (but not amyloid) SOD1 species to subsequently kill that specific mitochondrion.

As we seek to understand intricate details of the fibrillation/amyloid pathway of human SOD1 we are aware that effective therapeutic action may be best in order to avoid this pathway at all. An approach that appears promising involves stabilization of the dimer even when aberrant metalation is prevalent. Hasnain and co-workers have detailed a class of small molecules that bind Cys111 and shift equilibria away from monomerization and nucleus formation (Capper et al., 2018; Chantadul et al., 2020).

## Data Availability Statement

The original contributions presented in the study are included in the article/Supplementary Material, further inquiries can be directed to the corresponding author/s.

## Author Contributions

B-KK was first and major author. WM and EG provided help with some experiments. JV and JW directed research. JW was senior corresponding author. All authors contributed to the article and approved the submitted version.

## Conflict of Interest

The authors declare that the research was conducted in the absence of any commercial or financial relationships that could be construed as a potential conflict of interest.

## Supplementary Material

Supplementary Figure 1 Disulfide scrambling in apo WT-SOD1 (40 μM) at 0.5 mM TCEP. **(A)** TCEP concentration dependence of fibril formation for apo WT-SOD1. Fibrillation of WT-SOD1 in 10 mM potassium phosphate buffer, pH 7 is stimulated as higher TCEP concentration. TCEP concentrations are: 0 mM TCEP (green), 0.1 mM (navy), 0.2 mM (pink), 0.5 mM (cyan), 1 mM (gold), 5 mM (orange), and 10 mM (purple), respectively. **(B)** HPLC-MS spectrum of DTT reduced and iodoacetamide alkylated apo WT-SOD1 digested with trypsin. Each peak in the spectrum represents a tryptic peptide.

Supplementary Figure 2 LC-MS of apo AS-SOD1 fibril seeds produced from 5 mM TCEP in the presence of GdmCl **(A)** or no GdmCl **(B)**. Apo AS-SOD1 fibril seeds are completely reduced in the presence of GdmCl. Molecular weight of AS-SOD1: 15756.43, reduced and alkylated with IAA AS-SOD1: 15870.53.
