# Supplementary material for: A Novel SOD1 Intermediate Oligomer, Role of Free Thiols and Disulfide Exchange
Source: Front Neurosci. 2021 Feb 18;14:619279. doi: 10.3389/fnins.2020.619279 (PMC7930385; doi:10.3389/fnins.2020.619279)
Supplement: Supplementary Figure 1 — Disulfide scrambling in apo WT-SOD1 (40 μM) at 0.5 mM TCEP. (A) TCEP concentration dependence of fibril formation for apo WT-SOD1. Fibrillation of WT-SOD1 in 10 mM potassium phosphate buffer, pH 7 is stimulated as higher TCEP concentration. TCEP concentrations are: 0 mM TCEP (green), 0.1 mM (navy), 0.2 mM (pink), 0.5 mM (cyan), 1 mM (gold), 5 mM (orange), and 10 mM (purple), respectively. (B) HPLC-MS spectrum of DTT reduced and iodoacetamide alkylated apo WT-SOD1 digested with trypsin. Each peak in the spectrum represents a tryptic peptide. [file Data_Sheet_1.docx]

**Supplemental Figures:**


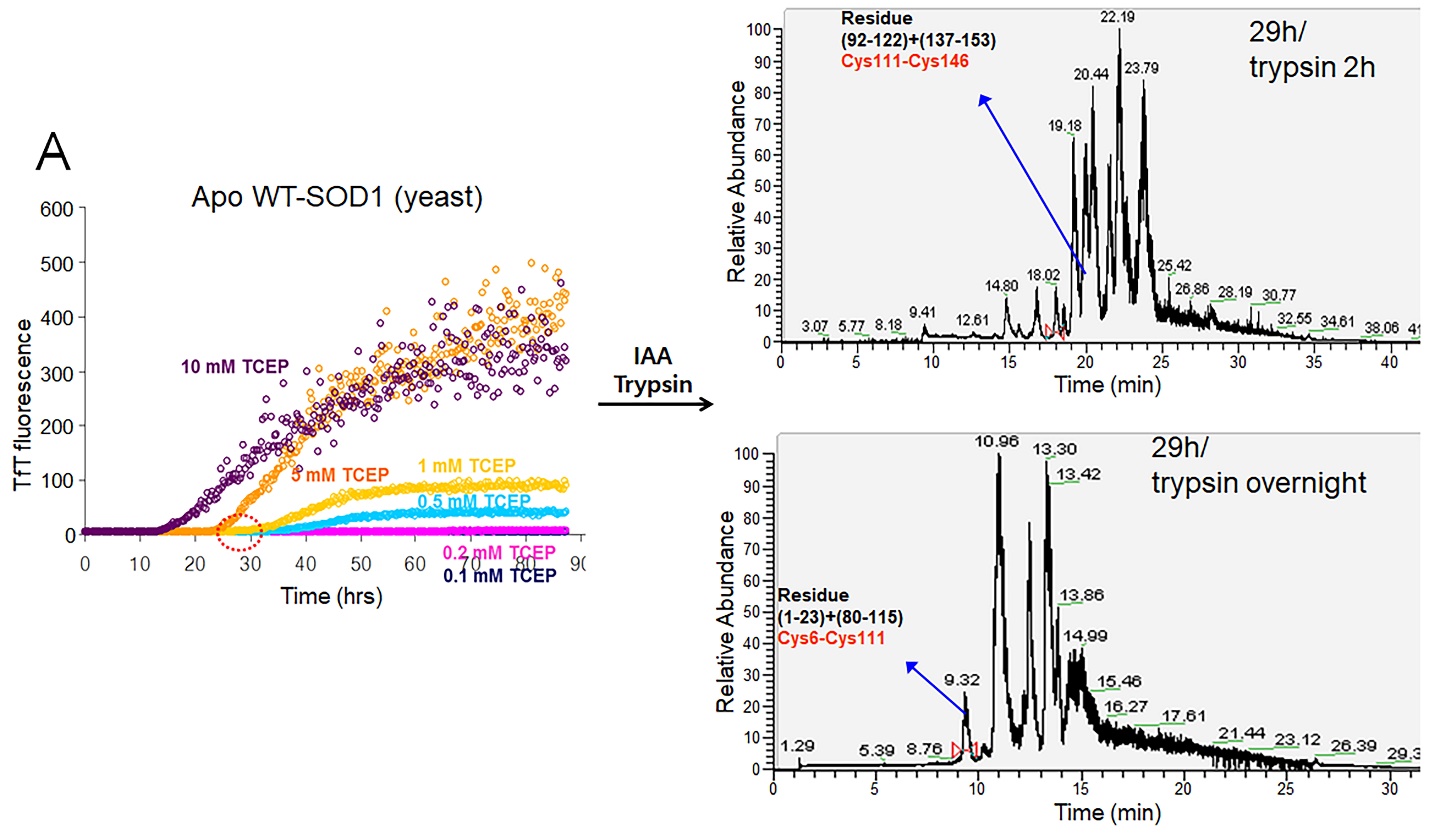


**Figure S1.** Disulfide scrambling in apo WT-SOD1 (40 µM) at 0.5 mM TCEP. (A) TCEP concentration dependence of fibril formation for apo WT-SOD1. Fibrillation of WT-SOD1 in 10 mM potassium phosphate buffer, pH 7 is stimulated as higher TCEP concentration. TCEP concentrations are: 0 mM TCEP (green), 0.1 mM (navy), 0.2 mM (pink), 0.5 mM (cyan), 1 mM (gold), 5 mM (orange), and 10 mM (purple), respectively. (B) HPLC-MS spectrum of DTT reduced and iodoacetamide alkylated apo WT-SOD1 digested with trypsin. Each peak in the spectrum represents a tryptic peptide.

**
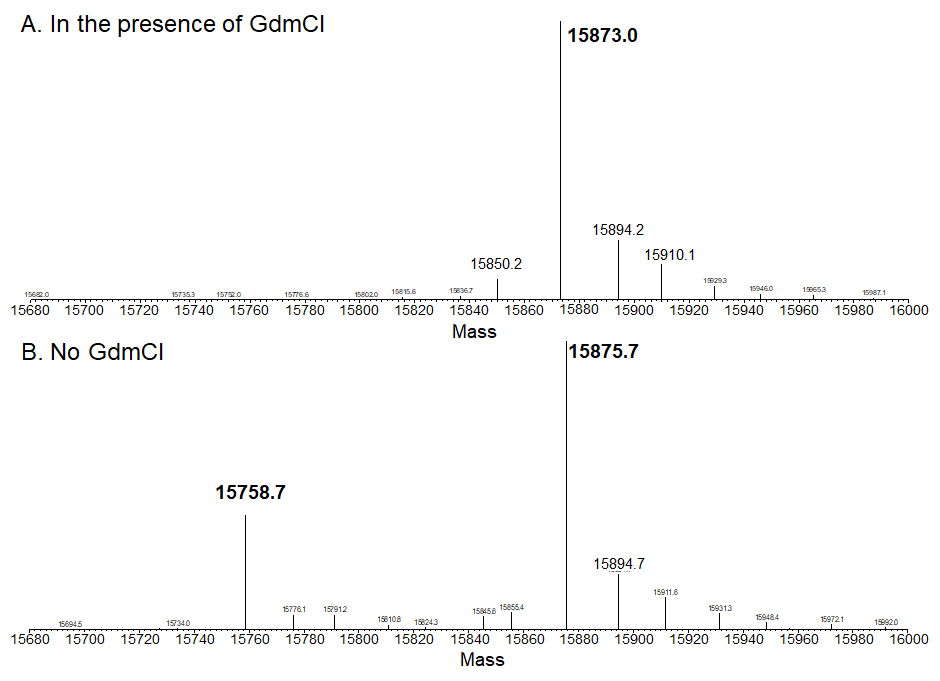
**

**Figure S2.** LC-MS of apo AS-SOD1 fibril seeds produced from 5 mM TCEP in the presence of GdmCl (A) or no GdmCl (B). Apo AS-SOD1 fibril seeds are completely reduced in the presence of GdmCl. Molecular weight of AS-SOD1: 15756.43, reduced and alkylated with IAA AS-SOD1: 15870.53.
